# Social inequities and dental caries in 5-year-old children: a study with results from SB Brasil 2023

**DOI:** 10.1590/1807-3107bor-2025.vol39.0046

**Published:** 2025-05-19

**Authors:** Rosa Núbia Vieira de MOURA, Saul Martins de PAIVA, Joana RAMOS-JORGE, Rafaela da Silveira PINTO, João Victor Inglês de LARA, Mariane Carolina Faria BARBOSA, Letícia Silva ALONSO, Andreia Maria Araújo DRUMMOND

**Affiliations:** (a) Universidade Federal de Minas Gerais – UFMG, School of Dentistry, Department of Social and Preventive Dentistry, Belo Horizonte, MG, Brazil.; (b) Universidade Federal de Minas Gerais – UFMG, School of Dentistry, Department of Child and Adolescent Oral Health, Belo Horizonte, MG, Brazil.; (c) Ministry of Health, General Coordination of Oral Health, Brasília, DF, Brazil.

**Keywords:** Child, Dental Caries, Health Services Research, Health Inequities

## Abstract

This study investigated the social inequities related to dental caries, clinical consequences of untreated caries, and the urgency to treat five-year-old children in Brazil. It is a cross-sectional study that used data from SB Brasil 2023 (n = 7198). The dmft index was used to assess dental caries, the *pufa* index to assess the pulp consequences of untreated caries lesions, and the need for treatment was identified. It included demographic and context variables: sex, color/race, enrollment in social programs, access to water in the household, and use of dental services. Logistic regression models for complex samples were used to estimate gross and adjusted odds ratio (OR), and confidence intervals (95%CI). The prevalence of early childhood caries (ECC) was 46.83%, with an average of 2.14 affected teeth, and 41.18% had untreated caries lesions. Non-white children and those enrolled in social programs had higher chances of having dental caries. The clinical consequences were associated with non-white children and with the last use of dental services being more than 3 years ago. Mothers with higher levels of education and the presence of piped water in the household were protective factors. This study highlights the high prevalence and inequities related to ECC in Brazil. Non-white children, beneficiaries of social programs, and those with limited use of dental services were more vulnerable to caries, its clinical consequences, and the urgency of immediate treatment. These results reinforce the importance of public policies to reduce inequalities and expand access to preventive dental care.

## Introduction

Early Childhood Caries (ECC) is a significant global public health problem, affecting approximately half of preschool-aged children.^1,2^ Studies indicate that, in addition to being highly prevalent, it is often untreated in children under three years of age.^3^ However, it causes serious economic and health burdens, resulting in a negative impact on the quality of life of the affected children and their families,^4-6^ including the disruption of caregivers’ daily activities, an economic burden on families and society, school performance issues, and difficulties in essential activities such as eating.^7^

The etiology of ECC is complex and multifactorial, strongly influenced by the health behaviors and practices of the child and their family. Additionally, structural factors and poorer socioeconomic conditions have a significant impact on the development of ECC, leading to inequalities that affect the unequal distribution of the disease in populations, with higher prevalence in low- and middle-income countries. Thus, although early childhood caries is preventable, it persists, reflecting generalized social and economic inequalities and inadequate funding for prevention and treatment actions.^1,3,7^

In Brazil, the implementation of public oral health policies has resulted in improvements in the oral health condition of the population, particularly in the reduction of caries prevalence in 12-year-old children. However, such advances have not been observed in primary dentition, and further investigation into the determinants of ECC is needed.^8,9^ In recent years, an unequal distribution of dental caries in five-year-old children has been observed across Brazil.^8,9^ The prevalence differences between regions were striking, with the North and Central-West regions showing the highest percentages (62.28% and 62.03%, respectively), and the Southeast region showing the lowest (36.72%).^9^

The World Health Organization recommends that prevention and control actions for early childhood caries be developed in an integrated manner with the primary healthcare systems and consider the effect of social determinants on ECC. Additionally, it advises that prevention efforts should focus on families and communities, including fluoride administration in both individual and collective strategies.^7^

Therefore, considering the relevance of early childhood caries for public health^3^ and the need to investigate the associated social determinants, this study aimed to assess the social inequities related to dental caries in early childhood, the clinical consequences of untreated caries, and the urgency for treatment in five-year-old children in Brazil.

## Methods

### Study design and population

This is a cross-sectional study conducted using secondary data from the National Oral Health Survey - SB Brasil 2023. The methodological procedures used in this survey, as well as its technical report, are available for online consultation.^9,10^ This study exclusively included children who were five years old, totaling 7,198 children. Of these, 13 did not have the clinical variables used in the analyses, so they were excluded from the sample. It is a representative sample at the national level, as well as for the macro-regions, states, and capitals .^10^

The SB Brasil 2023 Project was conducted in accordance with the ethical principles outlined in Resolution No. 466/12 of the National Health Council and was approved by the National Research Ethics Committee, under opinion 4.823.054. The Informed Consent Form (ICF) and the Informed Assent Form (IAF) were signed by all responsible individuals and children, respectively.

### Variables

The experience of dental caries was assessed based on the codes and criteria of the World Health Organization for evaluating the condition of the crowns of deciduous teeth (5 years old), and the dmft index (number of decayed deciduous teeth that had to be extrated and filled was calculated.^10,11^ The data were dichotomized into “Absence of carious lesions” (dmft = 0) and “Presence of carious lesions” (dmft ≥ 1).

To estimate the severity of caries and associated pathologies, the pufa index was used for the deciduous dentition, aiming to investigate the clinical consequences of untreated carious lesions.^12^. The presence of pulp involvement, ulceration, fistula, and dentoalveolar abscess were recorded.^10,12^ The data were dichotomized into “Absence of clinical consequences of caries” (pufa = 0) and “Presence of clinical consequences of caries” (pufa ≥ 1).

The need for dental treatment was recorded for each deciduous tooth according to the codes and criteria of the WHO Manual,^13^ with modifications suggested by the School of Public Health of the Universidade de São Paulo. The sum of the number of teeth examined with each of the assessed needs (no need/ need for one surface restoration/ restoration of two or more surfaces/ crown for any reason/ aesthetic facet/ pulp treatment and restoration/ extraction/ remineralization of white spot and sealant) was calculated. The data were dichotomized into “No need or need for preventive or routine treatment” and “Elective or immediate treatment”.

In addition to assessing oral issues, during the national epidemiological survey, a questionnaire was conducted with the participants’ guardians to collect demographic and socioeconomic information (sex [male; female], skin color/race [dichotomized in this study as white; non-white; don’t know/did not answer]), family information (participation in social programs [No; Yes; Don’t know/Did not answer], access to water in the household [Piped in at least one room; Piped only on the property or land; Not piped; Don’t know/Did not answer], and maternal level of education [re-categorized in this study as: Did not study/ adult education; Completed/Incomplete elementary school; Completed/Incomplete high school; Completed/Incomplete higher education; Don’t know/Did not answer]), and the use of dental services [Never went to the dentist; Up to 1 year; More than 1 to 2 years; More than 2 to 3 years; More than 3 years; Don’t know/Did not answer].^10^

### Statistical Analysis

For data analysis, the prevalence of dental caries in deciduous teeth, the clinical consequences of untreated carious lesions, and the need for dental treatment were considered dependent variables. Additionally, access to social programs, fluoridated water, sex, skin color/race, maternal level of education, and the use of dental services were considered independent variables. Descriptive analyses and logistic regression models for complex samples (n = 7185, with a loss of 13 participants whose clinical data were unavailable) were performed to estimate gross and adjusted odds ratios (OR) and their respective 95% confidence intervals (CI95%). Variables that presented p-values < 0.20 in the gross regression analysis were included in the adjusted logistic regression. The data was analyzed using SPSS Statistics 23.0 (IBM Corp., Armonk, USA).

## Results

The total sample of five-year-old children in project SB Brasil 2023 was 7,198 individuals. In this study, the sample comprised 7,185 children with complete data.

The prevalence of five-year-old Brazilian children who had experienced caries was 46.83%, with an average of 2.14 affected teeth. Notably, 41.18% of the children had one or more teeth with untreated carious lesions, with the decayed component representing 78.38% of the dmft index. Regional variations in dental caries experience (dmft ≥ 1) were observed across the states, with the lowest values in the Southeast (36.72%) and South (44.53%) regions, and the highest values in the North (62.28%) and Central-West (62.03%) regions. Regarding the presence of one or more teeth with untreated carious lesions, the highest values were found in the North (57.97%) and Central-West (52.03%) regions, and the lowest in the Southeast (31.30%) and South (36.82%) regions. In 14% of the children, at least one clinical consequence of dental caries was observed, with this prevalence ranging from 11.80% in the Southeast to 19.33% in the North.

Regarding the need for dental treatment in five-year-old children, 37.2% had never been to a dental appointment, and 45.4% had not sought dental services in the past year. Approximately 10% required urgent treatment due to pain, dental infection of oral origin, or referral for comprehensive evaluation or medical/dental treatment.

Descriptive analyses of sociodemographic variables, oral health, and treatment needs are presented in Table 1.

**Table 1:** Descriptive analysis of variables related to the dmft index, presence of clinical consequences and urgency of treatment in 5-year-old children in Brazil.

Variable	Total n	% (95%CI)	Patients with clinical data	dmft	PUFA	Urgency of treatment	
				Average (95%CI)	Average (95%CI)	No	Yes
						% (95%CI)	% (95%CI)
Child’s sex							
Male	3,670	48.9 (45.8–51.9)	3,670	2.17 (1.90–2.43)	0.31 (0.24–0.39)	88.0 (84.7–90.7)	12.0 (9.3–15.3)
Female	3,515	51.1 (48.1–54.2)	3,515	2.12 (1.86–2.38)	0.34 (0.25–0.43)	90.0 (87.3–92.1)	10.0 (7.9–12.7)
Skin color/Race							
White	2,490	43.2 (38.8–47.7)	2,488	1.63 (1.31–1.94)	0.22 (0.15–0.28)	91.2 (87.3–94.0)	8.8 (6.0–12.7)
Non–white	4,558	54.9 (50.4–59.2)	4,550	2.57 (2.32–2.81)	0.42 (0.32–0.52)	87.2 (84.3–89.6)	12.8 (10.4–15.7)
Doesn’t know/Did not answer	150	1.9 (1.1–3.4)	147	1.64 (0.86–2.41)	0.16 (0.05–0.27)	91.3 (84.3–95.4)	8.7 (4.6–15.7)
Mother’s level of education							
Did not study/ Adult education	659	9.3 (7.4–11.7)	658	2.06 (1.62–2.50)	0.35 (0.20–0.50)	86.4 (78.6–91.7)	13.6 (8.3–21.4)
Complete/Incomplete basic education	1,849	26.9 (23.4–30.7)	1,847	2.63 (2.25–3.01)	0.51 (0.32–0.70)	85.2 (81.2–88.5)	14.8 (11.5–18.8)
Complete/Incomplete high school	2,927	37.4 (34.5–40.4)	2,916	2.13 (1.79–2.46)	0.24 (0.18–0.30)	90.0 (86.8–92.5)	10.0 (7.5–13.2)
Complete/Incomplete highereducation	1,115	16.7 (12.9–21.5)	115	1.32 (0.90–1.74)	0.18 (0.06–0.29)	93.4 (87.5–96.7)	6.6 (3.3–12.5)
Doesn’t know/Did not answer	648	9.7 (7.6–12.2)	639	2.36 (1.71–3.01)	0.38 (0.16–0.60)	90.6 (82.0–95.4)	9.4 (4.6–18.0)
Social programs							
No	2,952	44.5 (39.2–49.9)	2,948	1.57 (1.31–1.83)	0.18 (0.12–0.23)	92.5 (89.6–94.6)	7.5 (5.4–10.4)
Yes	3,507	45.6 (40.9–50.3)	3,499	2.68 (2.38–2.99)	0.47 (0.36–0.59)	85.4 (82.3–88.0)	14.6 (12.0–17.7)
Doesn’t know/Did not answer	739	10.0 (6.8–14.5)	738	2.21 (1.53–2.88)	0.34 (0.14–0.54)	90.4 (83.6–94.6)	9.6 (5.4–16.4)
Water							
Piped, at least in one room	5,832	81.0 (76.4–84.8)	5,824	2.12 (1.87–2.36)	0.32 (0.24–0.40)	88.6 (85.9–90.9)	11.4 (9.1–14.1)
Piped only on the land/property	749	11.2 (8.4–14.9)	746	1.96 (1.52–2.40)	0.21 (0.10–0.31)	93.7 (89.6–96.3)	6.3 (3.7–10.4)
Not piped	172	2.9 (1.6–5.2)	171	3.89 (2.57–5.22)	1.12 (0.44–1.80)	79.1 (67.2–87.5)	20.9 (12.5–32.8)
Doesn’t know/Did not answer	445	4.9 (3.3–7.2)	444	1.97 (1.43–2.52)	0.28 (0.14–0.42)	91.3 (85.5–94.9)	8.7 (5.1–14.5)
Use of services							
Never been to the dentist	2,878	37.2 (33.2–41.3)	2,878	1.57 (1.30–1.84)	0.23 (0.15–0.31)	93.6 (90.8–95.5)	6.4 (4.5–9.2)
Up to one year	2,922	44.4 (40.8–48.1)	2,922	2.65 (2.25–3.06)	0.39 (0.25–0.53)	85.3 (80.6–88.9)	14.7 (11.1–19.4)
More than 1 year, up to 2 years	736	9.6 (8.1–11.3)	736	2.15 (1.68–2.62)	0.40 (0.21–0.59)	90.8 (86.2–94.0)	9.2 (6.0–13.8)
More than 2 years, up to 3 years	205	2.8 (1.6–4.7)	205	1.72 (0.61–2.62)	0.19 (0.05–0.32)	93.3 (85.8–97.0)	6.7 (3.0–14.2)
More than 3 years	129	2.2 (1.5–3.2)	129	2.54 (1.34–3.75)	0.67 (0.28–1.06)	69.7 (49.2–84.5)	30.3 (15.5–50.8)
Doesn’t know/Did not answer	315	3.8 (2.9–5.1)	315	1.80 (1.24–2.37)	0.21 (0.11–0.30)	89.0 (86.7–91.0)	11.0 (9.0–13.3)

Table 2 shows the results of the logistic regression analysis related to the presence of caries, clinical consequences, and urgency of treatment in five-year-old children in Brazil.

**Table 2:** Logistic regression related to the presence of caries, presence of clinical consequences, and urgency of treatment in 5-year-old children in Brazil.

Variable	Presence of caries		PUFA		Urgency of treatment	
	Experience of caries in one or more teeth (DMFT or dmft ≥ 1)		Presence of any tooth with clinical consequences of caries		No need/need for preventive or routine treatment/need for elective treatment X Immediate treatment/Referral (Systemic Condition)	
	OR Gross	OR Adjusted*	OR Gross	OR Adjusted*	OR Gross	OR Adjusted*
Child’s sex						
Male	ref	-	ref	-	ref	-
Female	0.91 (0.72–1.13)	-	0.99 (0.77–1.27)	-	0.82 (0.58–1.17)	-
Skin color/Race						
White	ref	ref	ref	ref	ref	ref
Non-white	**1.99 (1.55–2.57)**	**1.88 (1.47–2.40)**	**1.56 (1.06–2.30)**	**1.45 (1.01–2.08)**	1.53 (0.95–2.44)	1.42 (0.88–2.29)
Doesn’t know/Did not answer	1.19 (0.58–2.48)	1.21 (0.63–2.33)	0.75 (0.35–1.58)	0.72 (0.33–1.63)	0.99 (0.45–2.17)	1.22 (0.51–2.93)
Mother’s level of education						
Did not study/Adult education	ref	ref	ref	ref	ref	ref
Complete/Incomplete basic education	1.14 (0.75–1.73)	0.99 (0.67–1.48)	1.24 (0.64–2.40)	1.05 (0.54–2.05)	1.10 (0.57–2.14)	0.99 (0.50–1.94)
Complete/Incomplete high school	0.91 (0.62–1.33)	0.88 (0.59–1.31)	0.72 (0.43–1.21)	0.69 (0.40–1.21)	0.71 (0.43–1.15)	0.69 (0.41–1.15)
Complete/Incomplete higher education	**0.54 (0.36–0.81)**	**0.61 (0.40– 0.95)**	0.50 (0.17–1.44)	0.58 (0.19–1.78)	0.46 (0.18–1.14)	0.51 (0.19–1.39)
Doesn’t know/Did not answer	1.16 (0.69–1.95)	1.24 (0.71–2.14)	0.98 (0.44–2.19)	1.03 (0.42–2.51)	0.66 (0.25–1.71)	0.72 (0.24–2.13)
Income from social programs						
No	ref	ref	ref	ref	ref	ref
Yes	**2.04 (1.62–2.57)**	**1.84 (1.45–2.34)**	**2.22 (1.55–3.19)**	**1.97 (1.43–2.70)**	**2.10 (1.48–2.99)**	**1.98 (1.39–2.82)**
Does not know/Did not answer	1.65 (1.10–2.48)	1.54 (0.98–2.43)	1.71 (0.81–3.59)	1.90 (0.84–4.28)	1.30 (0.64–2.67)	1.37 (0.61–3.03)
Water						
Piped, at least in 1 room	ref	ref	ref	ref	ref	ref
Piped only on the land/property	1.07 (0.77–1.48)	0.87 (0.62–1.21)	**0.49 (0.29–0.81)**	**0.41 (0.24–0.69)**	**0.52 (0.28–0.95)**	**0.43 (0.25–0.76)**
Not piped	2.35 (1.17–4.72)	1.61 (0.65–3.99)	**3.18 (1.39–7.25)**	2.24 (0.90–5.60)	2.05 (1.05–3.99)	1.40 (0.67–2.92)
Doesn’t know/Did not answer	0.98 (0.68–1.42)	0.81 (0.49–1.32)	0.77 (0.44–1.36)	0.56 (0.37–1.19)	0.74 (0.40–1.36)	0.80 (0.40–1.57)
Use of dental service						
Never been to the dentist	ref	ref	ref	ref	ref	ref
Up to one year	**2.05 (1.47–2.86)**	**2.51 (1.87–3.36)**	**1.92 (1.15–3.19)**	**2.19 (1.38–3.46)**	**2.52 (1.48–4.29)**	**2.93 (1.77–4.84)**
More than 1 year, up to 2 years	1.57 (0.99–2.46)	1.73 (1.09–2.75)	1.51 (0.85–2.67)	1,46 (0,85–2,49)	1,47 (0,83–2,60)	1.52 (0.86–2.71)
More than 2 years, up to 3 years	0.67 (0.29–1.55)	0.80 (0.33–1.99)	1.13 (0.48–2.66)	1,22 (0,44–3,36)	1,05 (0,43–2,58))	1.11 (0.38–3.23)
More than 3 years	1.28 (0.58–2.81)	1.55 (0.65–3.70)	**3.95 (1.60–9.75)**	**4.79 (1.78–12.89)**	**6,35 (2,60–15,48)**	**7.49 (2.86–19.67)**
Doesn’t know/Did not answer	1.17 (0.70–1.95)	1.13 (0.67–1.91)	1.25 (0.64–2.41)	1.40 (0.63–3.08)	1,22 (0,61–2,45)	1.52 (0.64–3.59)

*Variables in bold: p < 0.05

Regarding the presence of caries and its contextual factors, non-white children had a higher likelihood of experiencing early childhood caries (OR = 1.88; 95%CI: 1.47– 2.40) and developing clinical consequences of untreated caries (OR = 1.45; 95%CI: 1.01–2.08). In terms of maternal level of education, children whose mothers had completed or partially completed higher education had a lower likelihood of experiencing early childhood caries (OR = 0.61; 95%CI: 0.40–0.95). Additionally, children from families whose income comes from social assistance programs were more likely to experience caries (OR = 1.84; 95%CI: 1.45–2.34) and to require urgent treatment (OR = 1.98; 95%CI:1.39–2.82).

For the clinical consequences of caries, the existence of piped water on the land or property was a protective factor. These children were less likely to show signs of clinical consequences of untreated caries (OR = 0.41; 95%CI: 0.24–0.69). Children who last used dental services more than three years ago had 4.79 times higher odds of exhibiting signs of clinical consequences of untreated caries (OR = 4.79; 95%CI: 1.78–12.89). Finally, children from families benefiting from social assistance programs were 1.98 times more likely to require immediate treatment/referral (systemic conditions) (OR = 1.98; 95%CI: 1.39–2.82).

## Discussion

This study analyzed the prevalence of early childhood caries, the clinical consequences of untreated caries, and the need for treatment in five-year-old Brazilian children in 2023. For the first time, social inequities related to the occurrence of caries were also investigated, considering possible contextual aspects of the disease. The results highlighted the existence of significant inequities that result in greater vulnerability to caries, its clinical consequences, and the need for immediate treatment among non-white children who are benefiting from social programs and have limited access to dental services.

Overall, monitoring the oral health conditions of the Brazilian population, through national surveys since the late 1980s, shows improvements in oral health, particularly in the reduction of caries experience.^8,14^ However, despite the reduction of caries in deciduous teeth of five-year-olds, the improvements have not been as significant as those observed in other age groups in Brazil. Recent data indicate that in 2003, 59.4% of five-year-old children had experienced caries (dmft = 2.8 teeth); in 2010, this number dropped to 53.4% (ceo-d = 2.4 teeth); and in 2023 to 46.83% (dmft = 2.14 teeth). However, the percentage of decayed teeth (untreated caries) in deciduous dentition remains high, being 84.2% in 2003, 80.3% in 2010, and 78.38% in 2023.^8,9,14^

These results demand extensive discussion to understand the contextual factors influencing the prevalence of caries and to contribute to the formulation of effective public policies. The Bangkok Conference, held in 2018, highlighted early childhood caries as a major public health issue. In addition to its high prevalence, the condition often remains untreated in children under three years old.^3^ In this study, 37.8% of five-year-old children needed elective or immediate treatment.

To improve access to treatment for this age group, it is essential to review and strengthen the National Oral Health Policy, known as Brasil Sorridente, including the mandatory implementation of preventive actions and treatments targeting the oral health needs of preschool-aged Brazilian children.^15^ It is worth noting that managing early childhood caries often requires extensive restorative interventions, extractions of deciduous teeth, and space maintenance. In cases of non-cooperative children, procedures involving sedation or general anesthesia may be necessary, leading to substantial costs.^3^

It is well known that dental caries, the presence of untreated caries, and its clinical consequences can negatively impact the quality of life of children and their families.^4-6,16^ In line with these findings, a Brazilian study demonstrated the positive impact of outpatient dental treatment on the oral health-related quality of life (OHQoL) of children and their families.^17^ Thus, it is crucial to expand the availability of dental treatment for this age group, with greater access to preventive consultations, aimed at reducing the incidence of untreated carious lesions and their clinical consequences, promoting improvements in the children’s quality of life. Additionally, public policies and programs focused on health promotion are essential for fostering citizenship and improving the quality of life and health of the population. One example is the Saúde na Escola (Health in Schools) Program, which aims to support the comprehensive education and citizenship development of students in basic education through partnerships between schools and primary healthcare units,^18,19^

Health can be understood from both macro and micro contexts. The macro context encompasses the social influences originating from family, community, and environment, shaped by community structures and policies. The micro context, on the other hand, covers biological, behavioral, and developmental aspects ^6,20^. The social pattern of health, diseases, and illness can be directly influenced by the social determinants of child health.^21^ Thus, to develop effective public policies aimed at reducing the prevalence of early childhood caries and improving the oral health-related quality of life (OHQoL) of preschoolers and their families, it is essential to recognize that oral health is influenced by factors at different levels: individual, family, and community.^3,22,23^

Early childhood caries is associated with a range of psychosocial risk factors.^3^ These include lower socioeconomic status, limited health literacy, frequent consumption of added sugar, increased bacterial plaque accumulation, and inadequate oral hygiene practices. Moreover, the educational level of caregivers and restricted access to fluoridated potable water are also directly linked to both the presence and severity of early childhood caries.^3,6,24-26^

Managing early childhood caries should begin before the onset of the disease through primary prevention strategies. Oral health education initiatives targeting parents and caregivers have proven effective in reducing ECC prevalence.^3,27^ Given that preschool-aged children rely on their caregivers for health care, it is essential to implement behavioral strategies that encourage the adoption and adherence to preventive practices.^27,28^ Furthermore, disease prevention requires fluoride exposure, both through collective methods like fluoridated public water supplies and individual methods such as fluoride toothpaste with a minimum concentration of 1,000 ppm.^3,29^

Another crucial aspect is implementing WHO guidelines on sugar consumption, which recommend avoiding sugar intake during the first two years of life and limiting added sugar consumption to less than 10% of the total caloric intake. It is also important to implement public policies that mitigate financial conflicts of interest involving the ultra-processed and sugary food and beverage industries.^30^

In this study, non-white individuals exhibited higher odds of experiencing dental caries, clinical consequences of untreated caries, and the need for dental treatment. Recently, a systematic review highlighted those individuals who self-identified as Black or Brown faced unfavorable oral health conditions, notably tooth loss, caries, and periodontitis.^31^

The relationship between racial inequities and oral health is associated with economic and social disadvantages, difficulties accessing adequate healthcare services, and discriminatory attitudes faced by the Black population.^32,33^ These inequities place non-white individuals in situations of social vulnerability, characterized by limited access to information and education, poorer working and housing conditions, lower purchasing power, and limited employment opportunities. These factors directly influence healthcare access and contribute to the perpetuation of inequalities.^31,34^

The National Oral Health Policy has implemented strategies to reduce oral health inequalities, including promoting fluoridation in the water supply nationwide, recognized as an essential health promotion measure.^35,36^ In this study, the presence of piped water, at least on the land, was identified as a protective factor. It is worth noting that fluoridation in public water supply is considered one of the ten greatest public health achievements of the 20th century, due to its proven effectiveness, high safety, and cost-effectiveness.^37^ Moreover, even in populations using fluoridated toothpaste, evidences indicate that water fluoridation remains effective in preventing caries in children.^38^

This study is relevant because it highlights social inequities related to early childhood caries and identifies risk factors such as non-white individuals, mothers with lower educational levels, and families whose income originate from social programs. These findings provide important insights for planning public interventions, allowing for more effective allocation of time and resources to groups with the greatest needs, contributing to the development of public policies that promote health equity. Furthermore, the study’s methodological robustness, conducted with a representative population sample, reinforces the validity and applicability of its results.

## Conclusion

This study found high prevalence and inequities related to early childhood caries in Brazil. Non-white children, beneficiaries of social programs, with limited use of dental services showed greater vulnerability to caries, its clinical consequences, and the need for immediate treatment. These results underscore the importance of public policies to reduce inequalities and expand access to preventive dental care. Such measures are essential to promote a more equitable and healthy future for Brazilian children.
